# Advanced oxidation processes (AOPs) for drinking water treatment: a state-of-the-art review on applications, efficacy, and implementation challenges

**DOI:** 10.1039/d6ra00697c

**Published:** 2026-04-28

**Authors:** Mohamed S. Attia, Mahmoud S. Abdel-Wahed, Faisal K. Algethami, Amer S. El-Kalliny

**Affiliations:** a Chemistry Department, College of Science, Imam Mohammad Ibn Saud Islamic University (IMSIU) Riyadh 11623 Saudi Arabia; b Water Pollution Research Department, National Research Centre 33 El Buhouth St., Dokki 12622 Giza Egypt msaadnew86@yahoo.com ms.abdel-wahed@nrc.sci.eg

## Abstract

Drinking water treatment plants (DWTPs) face challenges in upgrading their technologies to mitigate health risks and achieve environmental sustainability. Population expansion, limited water sources, and climate change all contribute to these difficulties. The primary reasons for rising water contamination are population growth, urbanization, and industrialization, as well as increased agricultural activities. Micropollutants (*e.g.*, pesticides, pharmaceuticals, industrial chemicals, *etc.*) are especially problematic because conventional treatment processes do not efficiently remove them. Advanced oxidation processes (AOPs), are required to improve drinking water quality in DWTPs since they are particularly successful at eliminating water pollutants. The purpose of this review is to provide an up-to-date and complete understanding of AOPs' use in drinking water treatment. It also attempts to close this gap by investigating the various forms of AOPs and their efficacy against various water contaminants such as natural organic matter, chlorination disinfection byproducts, and contaminants of emerging concern. Furthermore, this review will evaluate the practical implementation of AOPs, including their suitability for scaling up.

## Abstract 2

Drinking water treatment plants (DWTPs) face challenges in upgrading their technologies to mitigate health risks and achieve environmental sustainability.

## Graphical abstract

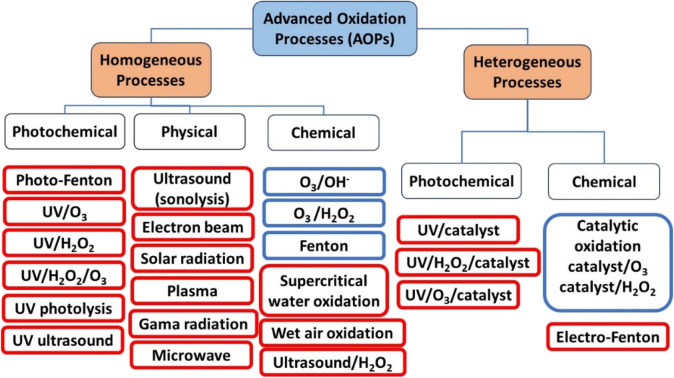

## 1. Introduction

The issue of global water scarcity has transcended geographical limits, thereby emerging as a significant challenge for the planet. This assertion is corroborated by estimates suggesting that, since the year 2000, more than 2.4 billion individuals have resided in countries facing water scarcity.^1^ Water scarcity is a pressing issue that currently affects approximately 40% of the global population. This issue is further compounded by the alarming rate of depletion of groundwater reserves, which is estimated to be between 100 and 200 km^3^ annually (United Nations, 2022). This deficit in quantity is exacerbated by serious quality concerns; for example, in severely stressed developing areas such as India, nearly 70% of the water supply is contaminated, contributing to the 2.2 billion individuals worldwide who do not have access to safely managed clean water. Consequently, these limitations necessitate a fundamental transition towards alternative water sources, including the reuse of wastewater, in accordance with Sustainable Development Goal 6.3, which focuses on enhancing water quality and treatment.^1^

Drinking water treatment plants (DWTPs) face challenges in optimizing their technologies in order to prevent health issues and ensure environmental sustainability.^2^ These challenges arise due to population growth, water source availability, and climate change.^3^ To disinfect the water, DWTPs primarily employ chlorination as it is a cost-effective means of deactivating pathogenic microorganisms.^4^ Chlorine is simple to dose, measure, and regulate. However, it cannot inactivate certain protozoa such as *Giardia lamblia* and *Cryptosporidium parvum*.^5^ Additionally, humic acids (HAs), which are the primary organic pollutants found in surface water, combine with chlorine to create chlorination disinfection byproducts (DBPs).^6^ These DBPs are thought to have carcinogenic properties.^5^ Moreover, contaminants of emerging concern (CECs), such as pesticides, pharmaceuticals, and personal care products, threaten human health and aquatic biota, thereby affecting the performance and costs of drinking water treatment technologies.^7^

Global contamination of water bodies with chemicals like pharmaceuticals, personal care products, and pesticides is a significant issue.^8^ This has been observed in many countries such as Chile, Australia, Romania, China, and Denmark. For instance, in Chile, agricultural activities near the Biobío River introduced these compounds into the aquatic environment. In Australia, 28 antibiotics were detected in six rivers and a water storage catchment.^9^ Due to their high chemical stability and limited biodegradability, conventional treatment methods are not effective in removing these chemicals. Therefore, advanced technologies, such as advanced oxidation processes (AOPs), are necessary to upgrade drinking water quality in DWTPs, as they are highly effective in removing water contaminants.^7^

AOPs are exploring the use of highly reactive radicals like hydroxyl radicals (˙OH) as a potential alternative for oxidizing various CECs and DBPs. Reactive nitrogen, sulfate, and chlorine radicals (Cl˙) can also play important roles.^10^ ˙OH are highly effective against chlorine-resistant protozoa such as *Cryptosporidium parvum* and *Giardia lamblia*.^5^ AOPs can be used independently, in combination with other AOPs, or in conjunction with traditional treatment methods depending on the wastewater stream's treatment objectives and features.^11^

In 1987, Glaze *et al.* coined the term “Advanced Oxidation Processes” to describe processes that generate enough ˙OH to purify water.^12^ Since then, the concept of AOPs has evolved to include a variety of techniques for producing reactive oxygen species such as singlet oxygen (^1^O_2_), superoxide anion radicals 
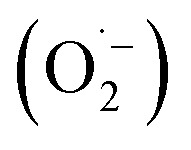
, and hydrogen peroxide (H_2_O_2_). However, ˙OH remains the most commonly associated species with AOP efficacy.^5^ The effectiveness of AOPs can be enhanced by using ultraviolet (UV) light and/or an effective catalyst. AOPs are a safe, versatile, and efficient water treatment method that can disinfect microbial agents, degrade contaminants, remove heavy metals, and oxidize taste-and-smell compounds, while minimizing emissions.^13^

**Fig. 1:**
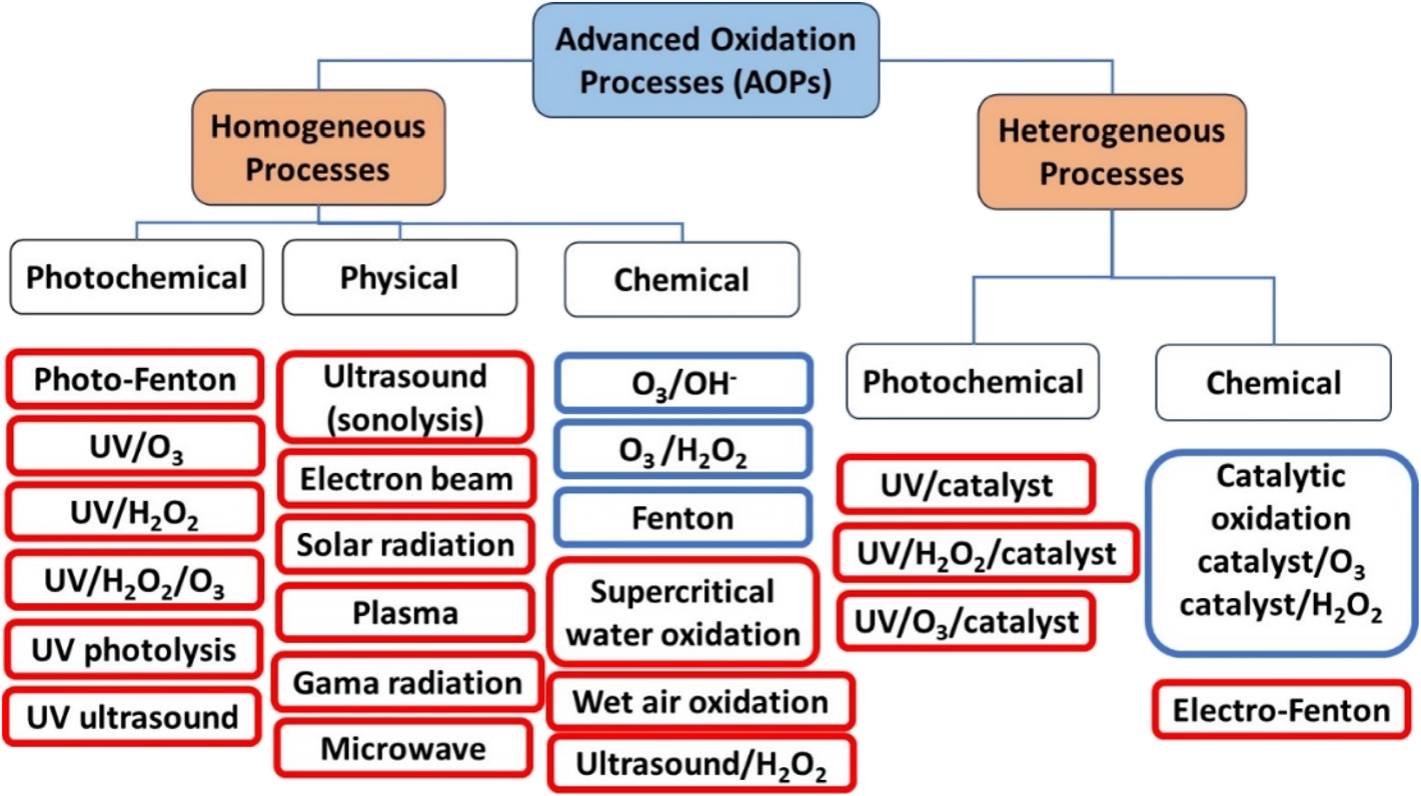
Classifications of different types of AOPs.

AOPs can be classified as either homogeneous or heterogeneous, as shown in Fig. 1.^14^ This classification is useful in distinguishing between different processes for scaling up. Homogeneous AOPs involve radical generators like ozone (O_3_) and H_2_O_2_, combined with oxidants like Fenton's reagent (H_2_O_2_/Fe^2+^) and photo-Fenton. Chemical homogeneous AOPs can occur through reactions involving Fenton or H_2_O_2_ and ultrasound. Physical homogeneous AOPs can occur through plasma, microwave or ultrasound. Photochemical homogeneous AOPs can occur through photo-Fenton or UV/O_3_. On the other hand, heterogeneity in AOPs is achieved through the use of semiconductors (catalyst). When a semiconductor is exposed to light of a specific wavelength, it generates reactive species that can oxidize organic molecules dissolved in water. It is also important to categorize AOPs based on whether they require energy or not. Processes that involve energy are indicated by a red frame in Fig. 1, while those that do not include energy are indicated by a blue frame. It is worth noting that most AOPs focus on energy-related processes, which makes their practical implementation more challenging.

### 1.1. Publication analysis

**Fig. 2:**
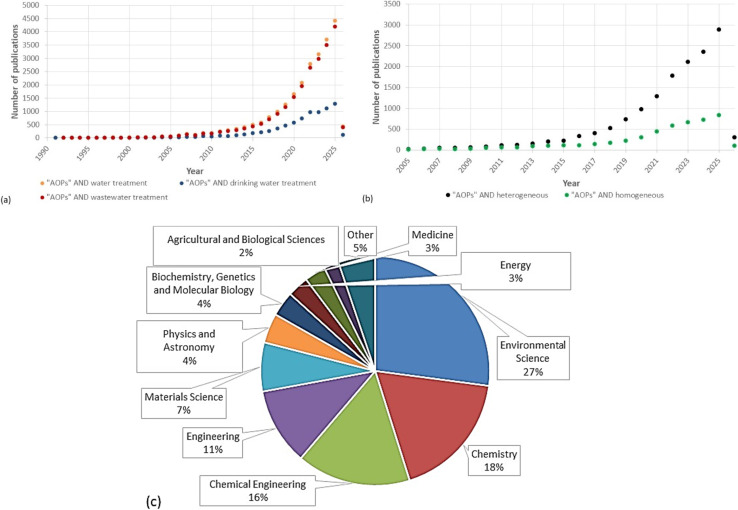
Comparison of the number of peer-reviewed publications. Data analysis of publications has been done using the Scopus scholar search system with the terms: (a) “AOPs and water treatment”, “AOPs and wastewater treatment”, “AOPs and drinking water treatment” from 1990 to January 2026, (b) “AOPs and heterogeneous”, “AOPs and homogeneous” from 2005 to January 2026, (c) the percentage of documents per subject area for “AOPs and water treatment” term.

As we delve into the applications of AOPs in drinking water treatment, it is worth noting that a survey has been conducted on peer-reviewed publications related to “AOPs and water treatment,” “AOPs and wastewater treatment,” and “AOPs and drinking water treatment” over the last 35 years. The results, as shown in Fig. 2a, indicate a significant increase in the number of publications in this field, primarily from 2005 onwards. There has been an exponential rise in AOP research publications in the past decade, which is due to the diversity of AOPs and their importance in removing CECs. Fig. 2a shows that the number of publications in the applications of AOPs for wastewater treatment is higher than for drinking water treatment. This indicates the importance of applying AOPs to remove CECs from the source point before reaching the DWTPs. Furthermore, there is an increasing trend in the number of publications and studies on using heterogeneous and homogeneous AOP systems. Fig. 2b shows that the number of publications on heterogeneous systems is higher than on homogeneous systems. This is due to the advantage of a heterogeneous system, where the catalyst can be separated from the treated water effluent, in contrast to a homogeneous system, where the precursor of the ˙OH and the reaction media are in the same phases. In addition to this, utilizing renewable energy sources, including solar photocatalysis, could lower treatment costs and increase the water industry's interest in AOPs. Fig. 2c shows the percentage of documents per subject area for AOPs in water treatment generally. The highest percentage of documents is for environmental science (27%), indicating that AOPs are potential techniques for environmental remediation. Comparable percentages of about 18% and 16% are for chemistry and chemical engineering, respectively, which show the attempts to increase the applicability of AOPs in water treatment. However, engineering only accounts for 11% of all research, which indicates that there are still challenges facing the scaling up of AOPs. Most of the research is focused on developing or introducing new applications for AOPs.

### 1.2. Novelty and objectives

AOPs have proven to be effective for water and wastewater treatment in several studies. However, their application in drinking water treatment requires further investigation to address concerns regarding hazardous oxidation byproducts, high energy consumption, and scaling up challenges. Fortunately, there are resources available that provide insight into AOPs' potential for drinking water treatment. While the foundational book by Gil *et al.* (2019)^5^ provides valuable insights into AOP mechanisms, this review offers distinct advancements by updating the state-of-the-art to encompass research from 2020–2026, including recent publication trends, emerging contaminants, and latest pilot-scale data such as the Siheung DWTP upgrades. Unlike previous descriptive works, this manuscript introduces practical quantitative decision support tools for engineers, specifically a Water Matrix Scavenging Capacity Framework, a Life-Cycle Assessment (LCA) scoring matrix, and a decision tree for AOP selection that link water quality targets to technology clusters. Furthermore, we provide a dedicated analysis of scalability and industrial usage to address the critical “lab-to-plant” gap by contrasting drinking water applications with mature wastewater implementations, alongside a comprehensive integrated sustainability assessment drawing on recent LCA literature (*e.g.*, Linden and Mohseni's 2014 chapter)^13^ to evaluate cost-effectiveness and environmental burdens holistically. By conducting this study, we aim to demonstrate the practical potential of AOPs in drinking water treatment, addressing implementation concerns to take a significant step towards improving water quality and ensuring access to safe drinking water for all.

However, there is still a need for an up-to-date, comprehensive understanding of AOPs' application in drinking water treatment. This study aims to bridge this gap by examining the different types of AOPs and their efficiency against various water contaminants, including NOM, DBPs, and CECs. Additionally, this study will assess the practical implementation of AOPs, including their availability for scaling up. By conducting this study, we hope to demonstrate the potential of AOPs in drinking water treatment and address concerns about their practical implementation. With this knowledge, we can take a significant step towards improving water quality and ensuring access to safe drinking water for all.

## 2. The limits of conventional drinking water technologies

Water treatment aims to prevent diseases, long-term health effects, and create palatable drinking water.^15^ Groundwater is mostly clean, but may contain compounds from biogeochemical processes and soil composition. Well conditions and surface water can also impact water quality. Karstic aquifers may be especially susceptible to surface water influence.^16^

**Fig. 3:**
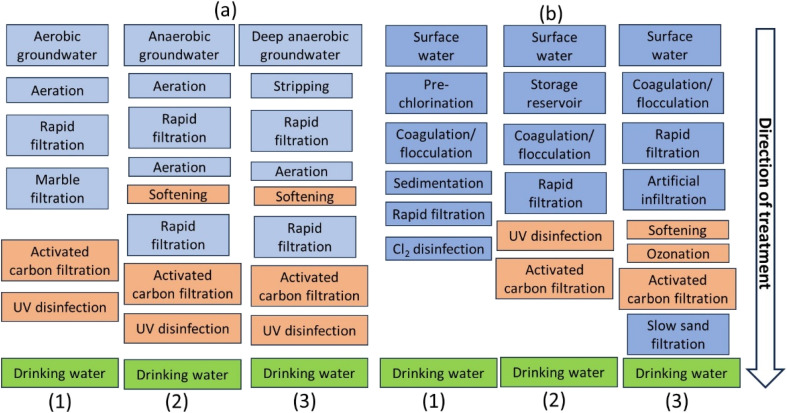
(a) Groundwater treatment schemes: (1) aerobic groundwater; (2) anaerobic groundwater, (3) deeply anaerobic groundwater. Orange blocks are optional, depending on the water quality: softening for hard water, activated carbon filtration and UV for the presence of organic micropollutants and disinfection. (b) Surface water treatment schemes: (1) traditional, (2) advanced, (3) advanced including soil passage.^18^

Groundwater can be aerobic, slightly anaerobic, or deeply anaerobic. Aerobic groundwater is open to the atmosphere and contains oxygen. To produce drinking water, it is aerated to increase O_2_ concentration and decrease CO_2_ concentration.^17^ Parameters like pH, calcium content, bicarbonate concentration, and saturation index are adjusted. Hard water, often in calcium-rich aquifers, shortens warm water device life and requires higher detergent concentrations.^18^ Groundwater types include aerobic, slightly anaerobic, and deeply anaerobic, with varying treatment requirements. Block schemes for typical groundwater treatment schemes are shown in Fig. 3a. Aerobic groundwater does not contain dissolved iron, while anaerobic groundwater contains dissolved iron, ammonium, and manganese. Aeration and stripping remove CO_2_ and oxidize Fe^2+^ to Fe^3+^, NH_4_^+^ to NO_3_^−^, and Mn^2+^ to MnO_2_, partly through chemical and biological processes. Fe^3+^ reacts with hydroxyl ions, forming Fe(OH)_3_ flocs. Bacteria convert ammonium, requiring oxygen. Deeply anaerobic groundwater contains high concentrations of iron, manganese, ammonium, hydrogen sulphide, methane, and chlorinated compounds from industrial contaminations. Aeration and stripping are used to remove gasses and oxidize these compounds, with dry filtration for nitrate formation.^17,18^

NOM in drinking water sources can negatively affect water quality and treatment processes, leading to performance issues, increased chemical use, and filter clogging. Hydrophobic NOM with high molar mass and aromatic carbon content is the primary cause of DBPs, causing reactions with bromine, iodine, and chlorine. Removing NOM is crucial for ensuring water supply safety.^19^

Organic micropollutants such as pesticides and pharmaceuticals have higher hydrophilicity and polarity than NOM, making flocculation less efficient.^20^ Activated carbon (AC) filtering has been increasingly used to eliminate these chemicals, as AC is highly efficient in eliminating hydrophobic organic molecules.^21^ However, NOM faces competition in eliminating micropollutants, obstruction of pores, reduced surface area for adsorption, and displacement of previously adsorbed compounds.^18^

Surface water is dynamic and contains pollutants, requiring treatment for suspended solids, disinfection, taste and odor compounds, and micropollutants.^21^ Traditional methods include coagulation, flocculation, sedimentation, and rapid filtration. Chlorination in some countries is no longer used due to health risks and DBPs.^22^ Alternative disinfection systems and multi-barrier approaches like O_3_ and AC filtration^23^ have been introduced (Fig. 3b). Traditional technologies remove macro parameters but have limited micropollutant removal performance. Advanced analytical technologies and changing compound use highlight emerging threats to drinking water quality.

Organic micropollutants can be removed through flocculation if they are already adsorbed to high molecular weight compounds. The pH level determines the charge and adsorption ability of these molecules.^24^ However, the removal of organic micropollutants by flocculation is minimal due to their solubility and hydrophilicity, as demonstrated by studies on pharmaceuticals by ref. 25 and pesticides by ref. 26. In addition, sweep coagulation and bridging remove bacteria and algae, while complexation with aluminum and iron removes viruses. However, these methods are insufficient for safe drinking water.^27^ Moreover, O_3_ is an effective disinfectant but can produce harmful byproducts. Membrane filtration may be an alternative but has drawbacks, such as fouling and leakage, that can reduce its effectiveness.^28^

UV irradiation is a highly effective method for deactivating microorganisms and cells, including bacteria, viruses, protozoan parasites, some spores, living cells, and subsystems such as enzymes, amino acids, and lipids.^18^ The use of UV for disinfection purposes dates back to 1901 in Marseille, but it wasn't until 50 years later that it was adopted on a larger scale in Switzerland, Austria, and Norway. Since the late 20th century, the use of UV for water disinfection has become very common.^18^ As shown in Fig. 3b, the introduction of UV systems as an advanced treatment for disinfection has paved the way for the widespread use of AOPs.

## 3. Homogeneous AOPs

Homogeneous AOPs dissolve precursors in water, resulting in free radicals reacting with water-soluble substances. They effectively remove NOM, especially dissolved organic carbon (DOC) that pass through filtration systems. Table 1 shows reduction percentages and mechanisms of producing ˙OH radicals.^29^

**Table 1:** DOC reduction by different AOPs with their respective mechanisms of producing ˙OH radicals^19^

AOP	Mechanism of ˙OH production^13^	DOC reduction^34^ (%)	Major system components^13^	Advantageous^13^	Disadvantageous^13^
O_3_/H_2_O_2_	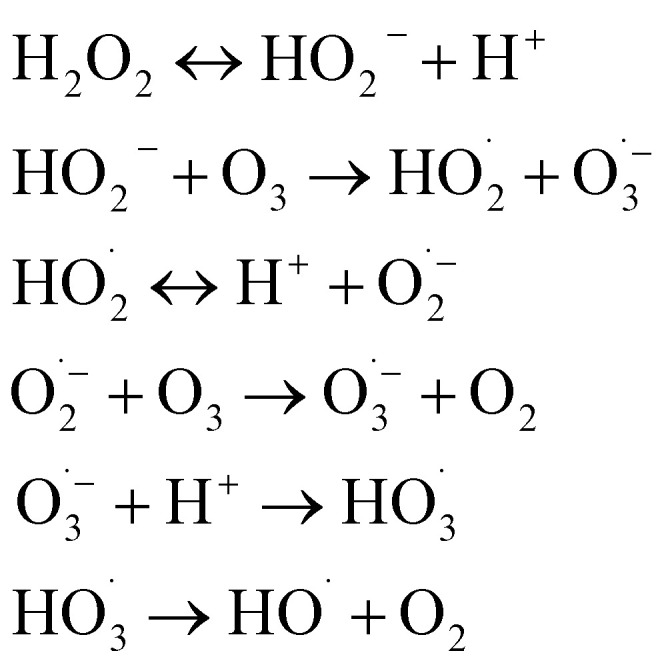	10–70	• H_2_O_2_ storage and injection system	• Established technology	• Potential for bromate formation
			• O_3_ generator, diffuser (static mixer), and contactor	• Efficient method for producing OH radicals	• May require quenching of excess/residual peroxide
			• O_3_ off-gas catalytic destructor	• Supplemental disinfection	• May require O_3_ off-gas treatment
			• Monitoring and control systems	• Effective at removing color and taste and odor	
UV/H_2_O_2_	H_2_O_2_ + *hν* (<300 nm) → 2HO˙	11–60	• UV lamp, ballast, sleeves, sensors, and lamp cleaning systems	• Established technology	• Not efficient for high turbidity and low transmittance water
			• H_2_O_2_ storage and injection system	• Supplemental disinfection	• Potential increase of DBPs (THMs and HAAs)
			• Monitoring and control systems	• No off-gas treatment required and no mass transfer limitation	• Requires quenching of excess/residual peroxide
O_3_/UV	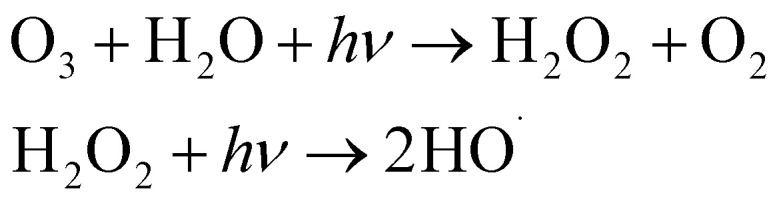	30–70	• O_3_ generator, diffuser (static mixer), and contactor	• Established technology	• Potential for bromate formation mass transfer limitation
			• O_3_ off-gas catalytic destructor	• Supplemental disinfection	• May require O_3_ off-gas treatment
			• UV lamp, ballast, sleeves, sensors, and lamp cleaning systems monitoring and control systems	• Effective at removing color and taste and odor	• Not efficient for high turbidity and low transmittance water
(O_3_/OH^−^)	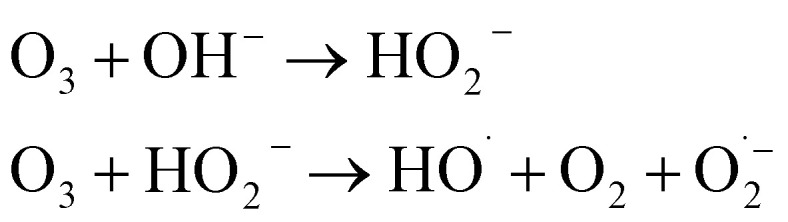	6–41	• O_3_ generator, diffuser (static mixer), and contactor	• Established technology	• Potential for bromate formation
			• O_3_ off-gas catalytic destructor	• Supplemental disinfection	• Mass transfer limitation
			• Monitoring and control systems	• Effective at removing color and taste and odor	• Requires pH adjustment
					• Requires O_3_ off-gas treatment
UV/TiO_2_	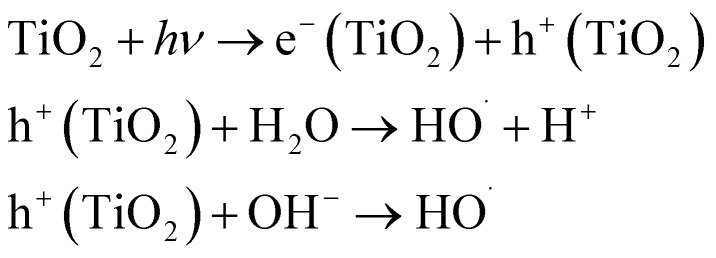	65–70	• TiO_2_ (slurry or coated on support)	• Could utilize higher wavelength (300–400 nm) irradiation	• Little full application success
			• Photocatalytic reactor	• Reagent free	• Low quantum yield
			• UV lamp, ballast, sleeves, sensors, and lamp cleaning systems monitoring and control systems	• No off-gas or no residual chemical treatment required	• Potential for rapid deactivation of photocatalyst
				• No bromate formation	• Requires downstream filtration if slurry photocatalyst is used
					• May require O_2_ sparging
					• Process would require close monitoring and control
Fenton	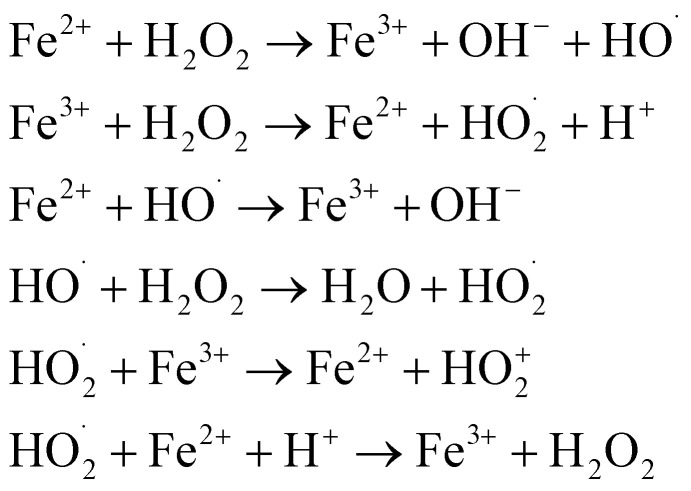	80–85	• Fe^2+^ and H_2_O_2_ storage and injection systems	• Established process with easy operation	• Low pH requirement
			• Stirred tank reactor	• No off-gas treatment required	• Require downstream sludge removal
			• Iron removal stage	• No bromate formation	• High operating cost because of pH adjustment and sludge removal
			• Monitoring and control systems		
Photo Fenton	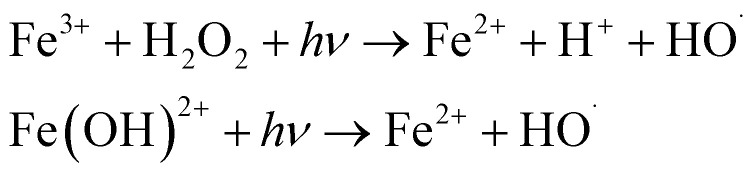	70–80	• Fe^2+^ and H_2_O_2_ storage and injection systems photoreactor	• *In situ* regeneration of Fe^2+^	• No full-scale application exists
			• UV lamp, ballast, sleeves, sensors, and lamp cleaning systems monitoring and control systems	• No off-gas treatment required	• Low pH requirement
				• No bromate formation	• High operating cost because of pH adjustment and UV lamp maintenance

**Fig. 4:**
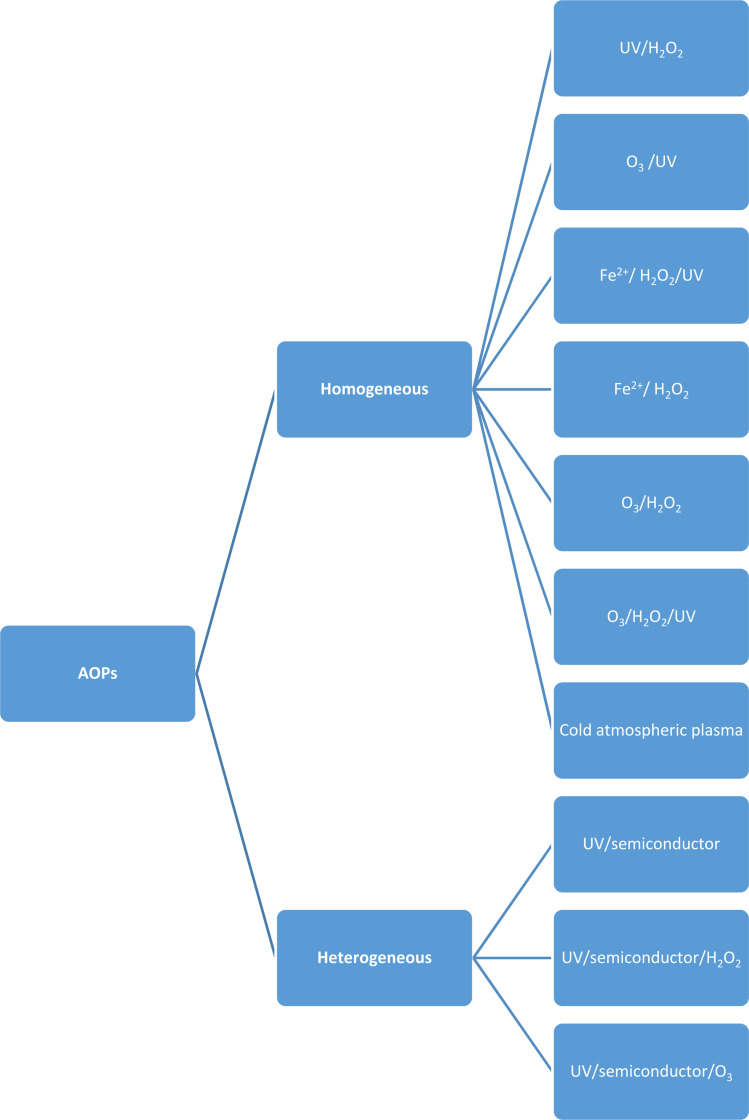
Schematic comparison figure summarizing homogeneous *vs.* heterogeneous AOPs.

Ozonation, ozone-based treatment (O_3_/H_2_O_2_), and Fenton (H_2_O_2_/Fe^2+^) are the most commonly used water treatment methods for chemical homogeneous AOPs. When combined with UV light, these methods become photochemical homogeneous AOPs and their potential effectiveness increases.^30^ During ozonation, O_3_ reacts with NOM by selectively adding to double bonds. On the other hand, ˙OH radicals are created from the reaction of O_3_ with water, but the potential for ˙OH radical formation is lower in ozonation than in AOPs. Combining O_3_ with UV light or hydrogen peroxide promotes ˙OH formation and reflecting to the higher treatment efficiency (see Table 1 and Fig. 4).

Ozonation processes operate through two distinct oxidation pathways: direct reaction with molecular O_3_ and indirect reaction *via* ˙OH generated during ozone decomposition. The selectivity and efficacy of the treatment depend heavily on the dominance of either pathway, which is governed by water quality parameters such as pH, dissolved organic matter (DOM), and inorganic scavengers.^31^

### 3.1. Direct oxidation

Molecular O_3_ is a selective electrophile (*E*^0^ = 2.07 V) that primarily reacts with organic compounds containing electron-rich functional groups, such as olefinic bonds, amines, and activated aromatics, *via* cycloaddition, electrophilic substitution, or oxygen atom transfer mechanisms. While effective for specific micropollutants, direct ozonation often results in partial oxidation, forming biodegradable organic byproducts rather than complete mineralization.^32^

### 3.2. Indirect oxidation (radical chain mechanism)

The non-selective indirect pathway involves the decomposition of ozone into hydroxyl radicals (*E*^0^ = 2.80 V), which react with organic contaminants at near diffusion-controlled rates (*k* ≈ 10^9^–10^10^ M^−1^ s^−1^). The decomposition mechanism is a chain reaction consisting of initiation, propagation, and termination steps.^32,33^

#### 3.2.1. Initiation

The chain reaction is typically initiated by the hydroxide ion (OH^−^), which reacts with ozone to form the hydroperoxyl anion (HO_2_^−^) and superoxide radical 
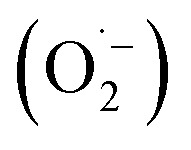
:

1 O_3_ + OH^−^ → HO_2_^−^ + O_2_ (*k* ≈ 70 M^−1^ s^−1^)

2
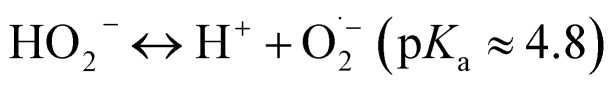


#### 3.2.2. Propagation

The superoxide radical anion 
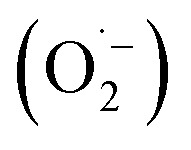
 reacts rapidly with another ozone molecule to form the ozonide radical 
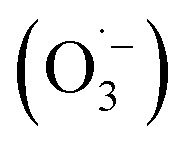
, which subsequently protonates and decomposes to yield the hydroxyl radical:3
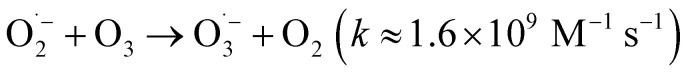
4
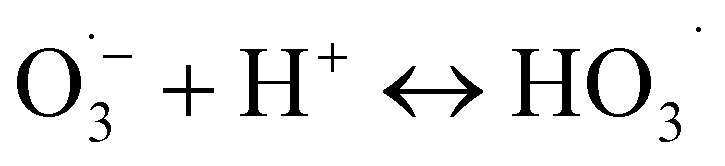
5
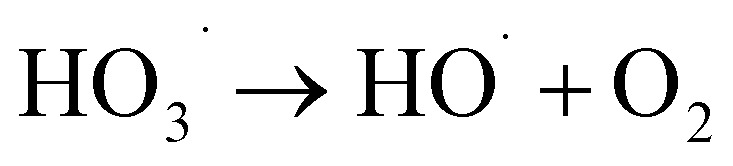


#### 3.2.3. Termination

The chain reaction is terminated when radicals are scavenged by species present in the water matrix. Bicarbonate (HCO_3_^−^) and carbonate (CO_3_^2−^) ions are potent scavengers that convert highly reactive ˙OH into the less reactive carbonate radical 
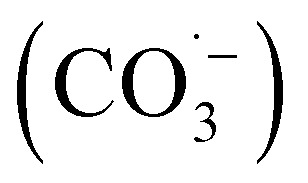
, thereby inhibiting pollutant degradation:6
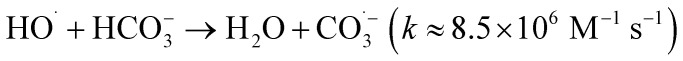
7
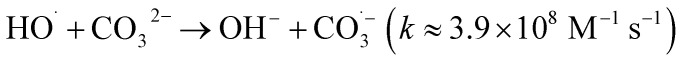


Consequently, enhancing ˙OH exposure often requires promoting ozone decomposition (*e.g.*, *via* high pH, UV irradiation, or H_2_O_2_ addition) while minimizing radical scavenging effects. This mechanistic understanding underpins the design of combined systems like O_3_/H_2_O_2_ (peroxone) and O_3_/UV, which accelerate the initiation step to maximize radical yield for recalcitrant contaminant removal.^31–33^

The impact of ozone on trihalomethanes (THMs) precursors is dependent on the type and composition of the organic material, which can have varying reactivities towards ozone and chlorine. Some studies have shown that ozonation modifies NOM, shifting it from a more reactive hydrophobic state that reacts quickly with chlorine to produce THMs, to a hydrophilic state that produces lower levels of THM.^35,36^ However, using of O_3_ can lead to the formation of bromate, a potential carcinogen, which is strictly regulated at 10 µg L^−1^ in drinking water. To minimize bromate formation, strategies include controlling bromide sources, optimizing ozone application, chemical adjustments (*e.g.*, adding ammonium, chlorine, or hydrogen peroxide), and pretreatment to lower bromide/DOM levels. Post-ozonation bromate removal is challenging, making prevention strategies critical. Given the complexity of bromate formation, tailored approaches considering both treatment goals and specific water quality parameters are necessary.^37^ Pulicharla *et al.* suggest that carboxylic acid byproducts (CABPs) from ozonation, which are present in higher concentrations, can encourage bacterial growth, potentially compromising water quality in distribution systems. They recommend implementing effective treatment processes to remove CABP precursors before chlorination is applied as a secondary disinfectant.^38^

According to research,^39^ combining O_3_ with H_2_O_2_ increases the total concentration of THMs compared to ozonated samples. Also, some studies^40^ showed no significant reduction in THMs after adding H_2_O_2_ or TiO_2_ to the ozone treatment. On the other hand, research^41^ found that O_3_/UV treatment resulted in decreased total organic carbon, trihalomethane formation potential (THMFP), and total organic halides compared to ozone alone. Additionally, O_3_/UV led to significant mineralization of DOC, lower THMFP, and haloacetic acid (HAA)formation potential.^42^ However, the combined system of O_3_/H_2_O_2_/UV was not remarkably more efficient than O_3_/UV in HAA decomposition.^43^

Reungoat *et al.*'s study found that ozone oxidation alone can remove 22 out of 25 micropollutants with efficiencies exceeding 85%. However, some contaminants, like gabapentin, roxithromycin, and caffeine, showed lower degradation rates.^44^ In other large-scale studies, the combination of ozone and hydrogen peroxide (O_3_/H_2_O_2_) was found to be even more effective in removing pharmaceuticals from wastewater reuse applications,^45^ generating ˙OH radicals that rapidly degrade a wider range of micropollutants.^13^

In a 2 years pilot-plant study, Scheideler *et al.* (2011) investigated the effectiveness of O_3_/H_2_O_2_ and UV/H_2_O_2_ AOPs in removing four micropollutants (atrazine, bromacil, ibuprofen, and *N*-nitrosodimethylamine (NDMA)) in surface water.^46^ The combination of ozone and hydrogen peroxide (at 2 g m^−3^ ozone and 5 mg L^−1^ H_2_O_2_) was found to provide excellent degradation of bromacil (>99%), relatively good removal of atrazine and ibuprofen (58% and 85%, respectively), and poor degradation of NDMA (approximately 9%). Incorporating UV (low-pressure UV reactors) following the O_3_/H_2_O_2_ system using UV doses ranging from 300 to 650 mJ cm^−2^ resulted in over 80% removal efficiency for all four micropollutants. The study concluded that the sequential application of O_3_/H_2_O_2_ followed by UV offers an optimal solution for effectively degrading micropollutant mixtures while optimizing energy consumption and minimizing the formation of harmful by-products such as bromate.^13^

Fenton's reagent in the dark has been used successfully to treat and/or pre-treat industrial wastewaters containing persistent organics. Photo improvement will be seen in the photo Fenton process. Irradiation with near-UV radiation and visible light improves the rate of organic pollutant removal and mineralization with the Fe^2+^/H_2_O_2_ and Fe^3+^/H_2_O_2_ reagents significantly. Irradiation has a beneficial influence on the degradation rate because it photo-reduces Fe^3+^ to Fe^2+^ ions, which produces fresh ˙OH and regenerates Fe^2+^ ions that can then react with the H_2_O_2_ molecules in the system (see Table 1). The Fenton process produces iron sludge because Fe^3+^ precipitates to iron oxyhydroxides, especially at higher pH. The resulting iron sludge must be removed, processed, and appropriately disposed of. Due to the low pH requirement and large iron sludge generation, Fenton and photo Fenton processes are not practical for drinking water treatment; thus, their uses have been limited to wastewater treatment.^13^ A critical limitation of the homogeneous Fenton process is the generation of substantial iron-containing sludge, primarily composed of ferric oxyhydroxides (FeOOH) and ferric hydroxides (Fe(OH)_3_), which precipitate during the mandatory pH neutralization step (typically pH 7–9) following acidic treatment (pH 2.5–3.5).^47^ This sludge production imposes significant operational burdens, including costs for dewatering, disposal, and compliance with hazardous waste regulations, which can account for up to 60% of total treatment expenses.^48^

It is worth to mentioned that the photolysis of hypochlorous acid (HOCl) and hypochlorite (OCl˙) generates reactive oxidants like ˙OH, Cl˙, and O_3_. The addition of light to chlorine disinfection units can transform traditional drinking water treatment systems into AOPs.^49^

Remucal C. K. and Manley D. (2016) critically assess previous studies on chlorine photolysis as a water treatment technology in a review article.^49^ They found that chlorine photolysis is capable to degrade model probe chemicals, organic pollutants, and to inactivate the chlorine-resistant bacteria. They discovered that the effectiveness of chlorine photolysis in producing reactive oxidants is depending on solution and irradiation conditions. Lower pH values lead to larger steady-state concentrations of ˙OH and Cl˙, which improves pollutant removal. They also discovered that, while the relative yields of DBPs during chlorine photolysis are also affected by solution conditions (for example, higher organic DBPs yields at low pH values), there is conflicting evidence about whether chlorine photolysis increases or decreases DBP production when compared to thermal reactions between chlorine and DOM in the dark. As a result, the pre-chlorination step in DWTPs using UV disinfection units may be dangerous if residual chlorine is present in the UV reactor.

**Fig. 5:**
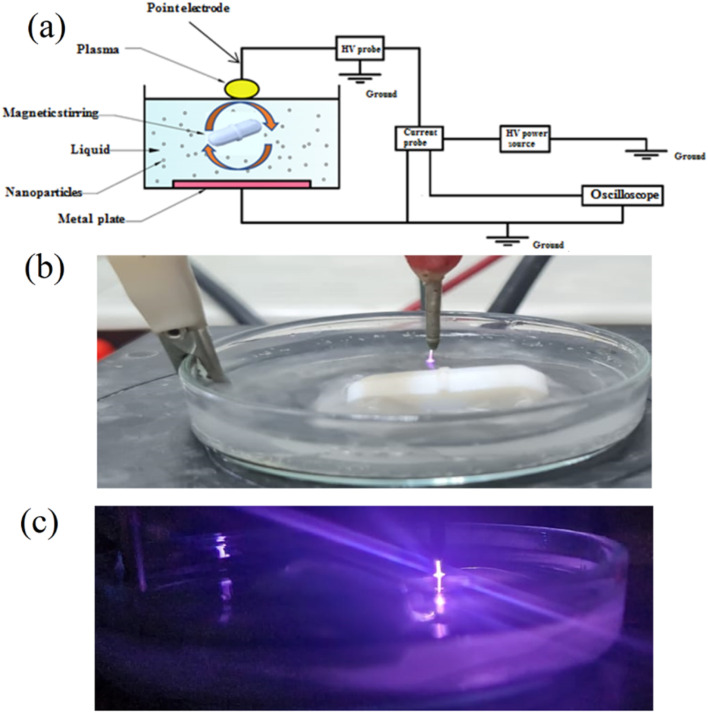
(a) Scheme of water treatment plasma system. (b) A photo of CAP source (point electrode). (c) A photo of the electrical discharge plasma in dark.^50^

Cold atmospheric plasma (CAP) has gained popularity in recent years due to its capacity to produce high densities of reactive oxygen species and reactive nitrogen species at room temperatures (Fig. 5). CAPs come in a variety of forms, with the most common being dielectric barrier discharges (DBD), DBD jets, radio-frequency controlled jets, and even microwave plasma sources. The bulk of these sources operate in a noble gas with a little amount of the molecular precursor. Some, however, can work in ambient air without the use of a gas source. Plasma-generated reactive species can be categorized into two broad categories: long-lived species such as H_2_O_2_, NO_2_^−^, and NO_3_^−^, and short-lived species, which can also initiate other chemical species in liquid, such as: O, ˙NO, ˙OH, 
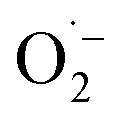
, and singlet molecular oxygen.^51^ Cold plasma technique provides speedy, low-energy, and successful cleanup for complicated polluted sites. Commercialization problems include upscaling and economic considerations. To establish an energy-efficient green cold plasma process, practical activities as well as long-term engineering and industrial requirements are required.^52^

## 4. Heterogeneous AOPs

Heterogeneous photo-oxidation involves a solid precursor producing free radicals at the contact between water and a catalyst, allowing for easier isolation from treated water effluent. The heterogeneous process for treating contaminated water offers advantages such as normal pressure and temperature, direct oxygen demand, inexpensive, safe, reuseable catalyst, immobilization on substrates, and solar light activation.^53^

In the aqueous phase, the procedure has five steps:^54^ the process involves transferring reactants from the bulk to the catalyst interface, adsorption on the catalyst surface, reaction on the adsorbed surface, desorption of products, and removal from the catalyst interface region.^55^ The brief mechanism of the producing ˙OH radicals for UV/catalyst (*e.g.* TiO_2_) is presented in Table 1.

The optimal photocatalytic performance is achieved when utilizing a photocatalyst with a small particle size in a slurry system. Solar light-based heterogeneous photo-oxidation shows promise as a water treatment solution. TiO_2_ has been extensively studied as a photocatalyst in this technology. This is due to its high oxidation power, moderate band gap, non-toxicity, and resistance to photo- and chemical corrosion.^56^

**Fig. 6:**
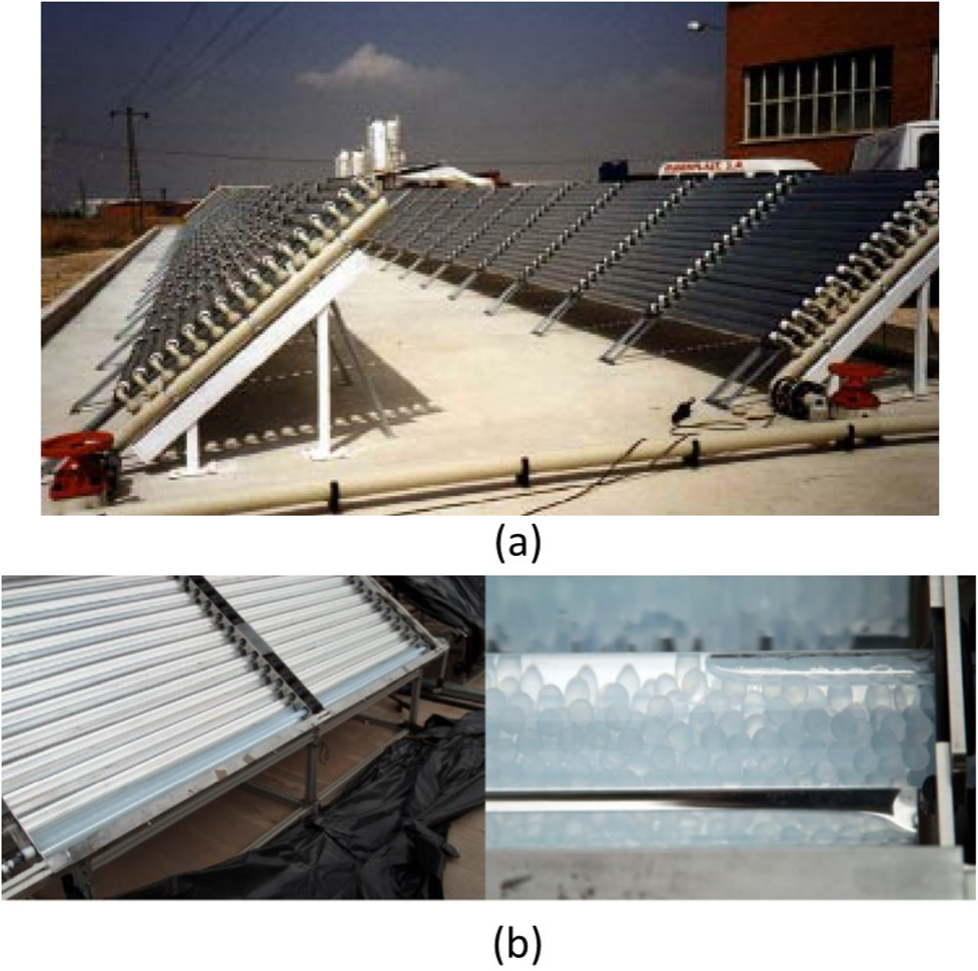
Pilot-plants based on CPCs: (a) TiO_2_ in slurry system,^57^ and (b) the tubes filled with the TiO_2_ coated spheres.^58^

For example, but not limited of pilot-plant design for slurry TiO_2_-solar system, construction, and testing, a full-size demonstration plant was built at HIDROCEN facilities in Madrid, Spain. Fig. 6a shows the compound parabolic concentrator (CPC) plant, designed to treat 1 m^3^ of water using 100 m^2^ of collector aperture area, uses anodized aluminum sheet and supports 16 parallel 1.5 m tubes. The final prototype plant has 21 collectors in parallel rows, connected in series using high dense polyethylene fast connectors. The facility operates automatically and requires minimal maintenance, showcasing its potential for commercial use.^57^

The extraction of photocatalyst from treated effluent is challenging, especially when used at nano-scale, limiting its practical implementation. Surface immobilization of catalyst particles addresses this issue, but may lower water oxidation potential due to limitations in mass transfer and light transport.^59^ Changes in reactor configuration can increase deterioration potential. Zhang *et al.* found that corrugated plates degraded 1.5 times faster than flat plates for the same reactor area.^60^ Complex arrangements improve catalyst surface to reactor volume ratio even more. Although the catalyst surface to reactor volume ratio may grow, reactor light penetration may limit it.^59^

**Fig. 7:**
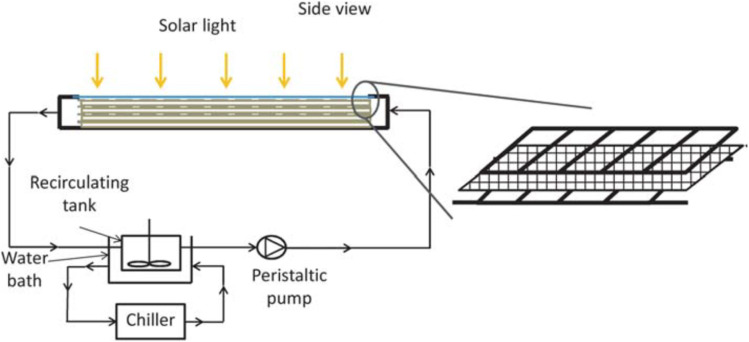
Schematic diagram of the fixed-bed solar photocatalytic reactor with a stack of TiO_2_ coated meshes.^65^

Stainless steel,^61^ borosilicate glass, ordinary and borosilicate glass,^62^ cellulose fibers,^63^ and other materials with diverse surface areas have been effectively coated with TiO_2_. The sol–gel dip-coating technique was employed to deposit the photoactive layer of TiO_2_ onto glass spheres for instance (Fig. 6b). The pilot CPC solar plant at the Plataforma Solar of Almeria was utilized to degrade fifteen emerging contaminants, each with an initial concentration of 100 µg L^−1^.^58^ In addition, a creative reactor with a stack of TiO_2_ coated meshes optimized active catalyst surface to reactor volume ratio, light penetration, and light distribution. El-Kalliny *et al.*^59,64^ found that four stainless steel meshes degraded HAs 3.4 times faster than a single plate flat-bed reactor (Fig. 7). This improved the photocatalytic efficiency of such reactors to that of dispersed-phase reactors without the TiO_2_ photocatalyst separation.

**Fig. 8:**
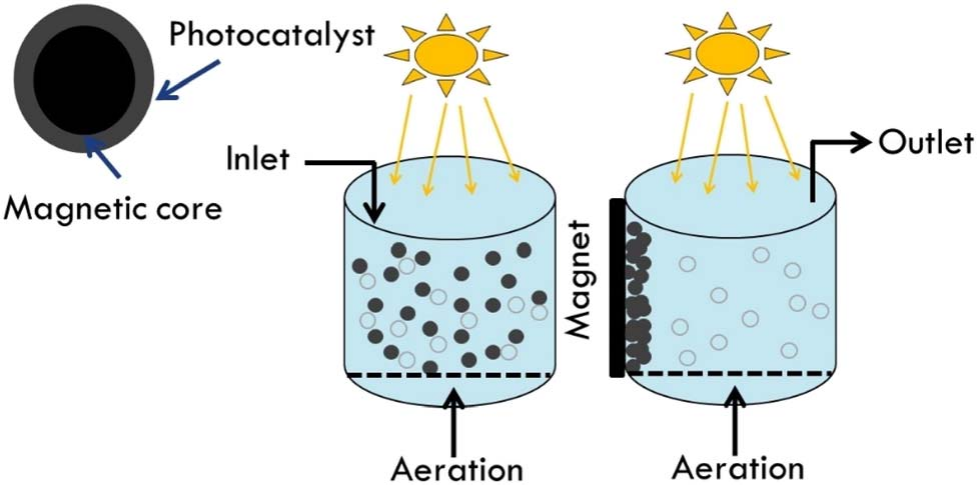
Schematic diagram for the processes of the magnetically separable photocatalyst.

Magnetic components can help separate, recover, and reuse photocatalyst from treated water effluents. External magnetic fields can easily separate TiO_2_ photocatalyst. In this scenario, TiO_2_ slurry boosts photocatalytic effectiveness due to its high active surface area. When employed as photocatalyst magnetic supports, spinel ferrites (MFe_2_O_4_, M = Ca, Mg, Zn, Co, Ni, Cd, *etc.*) outperform magnetite Fe_3_O_4_ due to their thermal and chemical stabilities. Ferrites can be superparamagnetic, with low retentivity. This low retentivity is favored in water treatment because magnetic particles gather solely by external magnetic field and disperse well without losing surface area. In addition using solar light is cost effective (Fig. 8).^56^

A 2020 review by Jacinto *et al.* covered magnetic materials for photocatalysis.^66^ This study examined single-phase catalysts, composites, multifunctional metal–organic framework materials, binary and ternary core magnetic shell, and yolk–shell photocatalysts. Most magnetic separable photocatalysts degrade water pollutants well. Sciscenko *et al.* used Magnox, magnetic SiO_2_/Fe_3_O_4_ covered with TiO_2_, for pilot plant tertiary wastewater disinfection and enrofloxacin abatement.^67^ However, to the best of our knowledge there is no pilot plant scale for drinking water treatment using magnetic photocatalyst. This may be related to reactor light distribution or electromagnetic unit efficiency issues.

### 4.1. Catalyst stability, recyclability, and regeneration

In the context of drinking water applications, the catalyst's stability is of paramount importance, not only from an economic perspective but also from a safety perspective.^68^ Mixed metal oxides and spinel ferrites (for example, CoFe_2_O_4_, MnFe_2_O_4_) typically exhibit superior chemical stability due to their robust crystal lattice structures, which minimize metal leaching over extended operation periods.^69^ Recyclability is frequently measured by the retention of degradation efficiency across successive cycles. The utilization of magnetic ferrites is gaining popularity due to their stability and magnetic separability. This property enables straightforward recovery and reuse without the need for filtration, which can result in significant losses.^70^ Whilst titanium dioxide (TiO_2_) and zinc oxide (ZnO) have been the focus of much research, these materials frequently exhibit aggregation tendencies or necessitate immobilization on supports, such as activated carbon or graphene, to ensure their recyclability.^71^ In contrast, metal sulphides (*e.g.* CdS, ZnS) demonstrate excellent visible-light absorption; however, they are susceptible to photo-corrosion (self-oxidation by photogenerated holes), resulting in rapid structural degradation and reduced durability in comparison to oxide counterparts.^72^ When catalytic activity declines, it may be due to surface fouling or intermediate adsorption. In such cases, the implementation of regenerative techniques becomes imperative.^73^ Chemical washing is defined as the process of using solvents, such as ethanol or NaOH, to desorb accumulated by-products. It contributes to operational costs and the energy footprint, emphasizing the need for future research to develop inherently durable catalyst designs.

## 5. Selection of AOPs

Choosing the most efficient AOP for treating contaminated water can be challenging due to various factors, including the type of contaminant(s) and the water matrix.^74^ Comparing AOPs can also be difficult, given the differences in the fundamental processes used for generating hydroxyl ˙OH radicals. However, with a good understanding of these factors, it's possible to select the most optimal AOP for your specific needs and achieve superior results. Therefore, selecting the appropriate AOPs, certain criteria need to be considered. These criteria will be briefly discussed below.^13^

### 5.1. Selecting based on efficiency

It is an undeniable fact that the efficiency of AOPs, the advanced oxidation processes, depends on several factors. The type of contaminant, its concentration, water matrix, constituents, reactor configuration, and design all play a crucial role in determining the success of AOPs.^75^ To make a fair and meaningful comparison between the effectiveness of AOPs, all these variables must be thoroughly considered. However, controlling these factors is often a challenge, making the use of AOPs all the more important.

### 5.2. Selecting based on electrical energy

AOPs used for photodegradation can be energy-intensive, with electrical energy representing a significant portion of operational costs. To compare electrical energy efficiency, figures-of-merit have been developed.^76^ The most common figure-of-merit is the electrical energy per order (*E*_EO_), which measures the electrical energy required to remove 90% of contaminants in 1 m^3^ of contaminated water. *E*_EO_ (kWh per m^3^ per order) values are calculated using a specific formula as follows:8
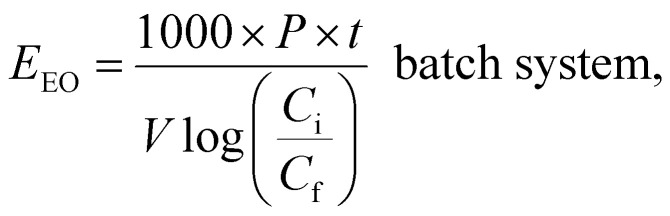
9
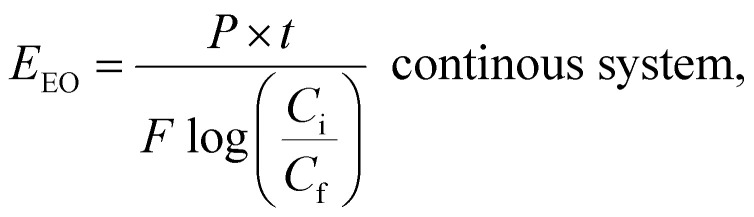
where *P* (kW) is the power of the system, *V* (L) is the water volume, *t* (h) is the treatment time, *C*_i_ and *C*_f_ in mol L^−1^ are the initial and the final concentrations of the water contaminant, and *F* (m^3^ h^−1^) is the flow rate in the continuous flow-through reactor. *E*_EO_ can be influenced by various factors, such as the quality of the water (including its matrix and constituents), the flow rate, the characteristics of the contaminant, and the design of the reactor.

As illustrated in Table 2, a thorough comparison of *E*_EO_ values for a range of AOPs is presented, providing fundamental insights into their energy efficiency for contaminant degradation. The data indicates substantial variations in energy requirements among different AOP types, with *E*_EO_ values ranging from 0.07 kWh per m^3^ per order to 8000 kWh per m^3^ per order. It is evident from the analysis that traditional ozone-based processes demonstrate superior energy efficiency, with ozonation showing the lowest median *E*_EO_ value of 0.15 kWh per m^3^ per order (range: 0.07–0.3), followed by O_3_/H_2_O_2_ processes with a median of 0.2 kWh per m^3^ per order. It is noteworthy that electron beam (EB) technology demonstrates competitive performance with a median *E*_EO_ of 0.3 kWh per m^3^ per order (range: 0.2–1), thus positioning it among the most energy-efficient AOPs available. It is evident that UV-based processes demonstrate moderate efficiency, with UV/H_2_O_2_, O_3_/UV, and UV/persulfate exhibiting median *E*_EO_ values ranging from 0.4 to 0.75 kWh per m^3^ per order. However, processes such as photo-Fenton (median: 2.6), plasma treatments (median: 3.3), and particularly ultrasound (median: 2600) and microwave (median: 540) technologies demonstrate substantially higher energy requirements, indicating their limited practical applicability for large-scale water treatment applications. The selection of technology must also consider factors beyond energy consumption, including operational costs, maintenance requirements, and treatment effectiveness for specific contaminants.^76–78^

**Table 2:** Reports concerning the *E*_EO_ values established for various AOPs^77^

Type AOPs	*E*_EO_ range [kWh per m^3^ per order]	*E*_EO_ median value [kWh per m^3^ per order]
UV/H_2_O_2_	0.25–1	0.7
Ozonation	0.07–0.3	0.15
O_3_/H_2_O_2_	0.1–1.5	0.2
UV/Cl	0.2–0.7	0.4
UV/persulfate	0.2–1.1	0.67
UV/H_2_O_2_	0.3–1.2	0.75
Photo-Fenton	1.1–10	2.6
Plasma (any)	1.1–12	3.3
UV/catalyst	150–450	335
Microwave	500–700	540
Ultrasound	800–8000	2600

### 5.3. Selecting based on reliability

Reliability is critical when choosing a technology for a specific application. Established AOPs like O_3_/H_2_O_2_, UV/H_2_O_2_, and UV/O_3_ are reliable and meet treatment goals consistently. Other AOPs like UV/TiO_2_ photocatalysis, catalytic ozonation, and Fenton's reactions may not be suitable due to their untested nature in large-scale applications and potential for catalyst deactivation. Mechanical reliability is also crucial in comparing different processes. For instance, O_3_/H_2_O_2_-based AOP is the most mechanical reliable technology as it requires less maintenance. The UV-based AOPs and Fenton's processes are less reliable due to their need for catalyst addition and significant operational and maintenance oversight. Therefore, it is necessary to consider reliability when selecting a technology to ensure it meets treatment goals under specific conditions.^13^

### 5.4. Selecting based on robustness

Robustness means maintaining consistent performance despite variable operating conditions like flow rate and water quality. A good treatment process should handle wide fluctuations in the inlet water flow rate without affecting the process outcome. AOPs like O_3_/H_2_O_2_, UV/H_2_O_2_, and UV/O_3_ are considered robust when safety factors are incorporated. These methods allow for chemical dosing or UV flux adjustment to suit changing flow rates. UV-based AOPs can also be designed modularly to handle large flow rates and high contaminant concentrations. Water quality factors like alkalinity, turbidity, and background NOM can impact AOP performance. Radical scavengers like alkalinity and NOM can hamper treatment performance as ˙OH plays a crucial role in AOP effectiveness. Other factors like turbidity, nitrate, and inorganic ions such as iron can also reduce ozone- and UV-based AOPs' efficiency. These parameters are considered during the design stage to ensure treatment performance is maintained within the expected water quality ranges.^13^

### 5.5. Ease of implementation

AOPs can be easily incorporated into a treatment train. Modular processes like UV/H_2_O_2_, VUV, and UV/TiO_2_ are more adaptable than non-modular systems like ozone-based AOPs. They offer flexibility in residence time and chemical dosage.^13^ However, some AOPs require additional pre- or post-treatment systems, which can add to the cost and hinder the implementation of the process. For example, Fenton's reactions require post-treatment to remove iron, making them less viable for drinking water applications. Similarly, UV-based oxidation processes often need pre-treatment to reduce turbidity, NOM, nitrate, and other inorganic ions, which can affect UV transmittance. Oxidant residuals like ozone or hydrogen peroxide may also need to be quenched before downstream treatment.

**Fig. 9:**
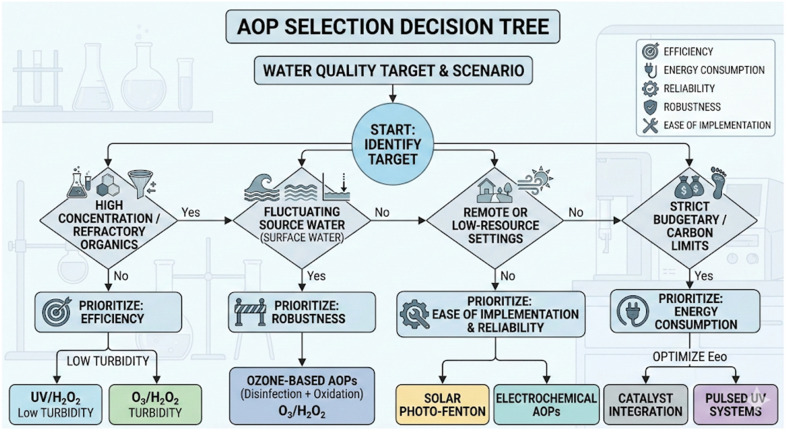
A strategic decision-making framework for selecting AOPs in drinking water treatment.

Fig. 9 shows a strategic decision-making framework for selecting AOPs for drinking water treatment. It shows how to prioritize the aforementioned five criteria for AOP selection (efficiency, energy consumption, reliability, robustness, and ease of implementation) based on the specific water quality and resource limits at each site. The decision tree is a systematic screening tool that matches the characteristics of the water influent and operational limits with the AOP configuration that is technically most feasible. The framework first helps the user determine which water contaminants they are dealing with. Then directs them to focus on efficiency for refractory organic compounds or robustness for fluctuating surface water sources, where multi-barrier protection (disinfection and oxidation) is needed. When geographic isolation or limited funds are an issue, the focus shifts to systems that are easy to set up and use less energy, such as solar-powered processes or pulsed UV. This hierarchical approach ensures that the selected technology is not just a high-performance lab solution but also a long-lasting, adaptable application that meets the treatment facility's socio-technical needs.

## 6. Water matrix effects

The efficiency of AOPs is highly dependent on water quality parameters, as matrix components can act as radical scavengers, compete for oxidants, absorb UV light, or form inhibitory complexes. Understanding these effects quantitatively is critical for process design and optimization. Recent comprehensive reviews have synthesized extensive data on matrix effects in photocatalytic and AOP systems, which we integrate here for drinking water applications.^79^

• Chloride ions: generally inhibitory across most AOPs by forming less reactive chlorine radicals (Cl˙, 
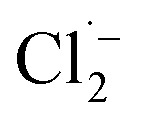
); effect magnitude depends on concentration and catalyst type.^79^

• Bicarbonate/carbonate: consistently inhibitory due to rapid ˙OH scavenging, forming less reactive carbonate radicals 
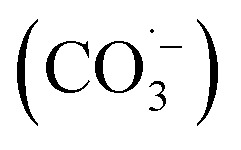
; particularly impactful for UV-based AOPs.^79^

• Phosphate: strongly inhibitory for heterogeneous photocatalysis (TiO_2_, g-C_3_N_4_) due to competitive adsorption blocking active sites.^79^

• NOM: dual role can act as electron donor improving charge separation at low concentrations, but predominantly inhibitory at typical drinking water levels *via* scavenging, light absorption (inner filter effect), and competitive adsorption.^79^

### 6.1. Influence of physicochemical parameters

Beyond chemical constituents, physical water parameters significantly modulate AOP performance:^79^

• pH effects: pH influences catalyst surface charge (relative to point of zero charge, PZC), pollutant ionization state, and ROS generation pathways. For TiO_2_ (PZC ≈ 6.8), acidic conditions favor adsorption of anionic pollutants but may increase electron–hole recombination; alkaline conditions promote ˙OH formation from OH^−^ but may repel anionic targets.^79^ Optimal pH is pollutant- and catalyst-specific, typically neutral to slightly alkaline (pH 6–8) for drinking water applications.

• Temperature: elevated temperatures generally increase reaction kinetics but may accelerate electron–hole recombination and reduce ROS stability. Moderate temperatures (25–35 °C) often provide optimal balance for photocatalytic systems.^79^

• Dissolved oxygen (DO): critical electron acceptor preventing charge recombination and enabling superoxide radical 
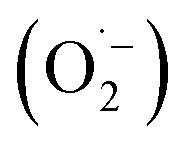
 formation. Higher DO levels generally enhance photocatalytic efficiency; oxygen-deficient conditions significantly reduce degradation rates.^79^

• Turbidity and suspended solids: reduce light penetration *via* scattering/absorption, compete for adsorption sites, and can foul catalyst surfaces. Pre-filtration is often essential for UV-based and photocatalytic AOPs in turbid waters.^79^

## 7. Financial considerations

The energy requirement and cost of AOPs for drinking water treatment are site-specific and influenced by target compounds, water quality, and UV transmittance. Most studies have focused on operating costs, but with AOPs increasingly used in large-scale applications, more complete economic data is expected.^13^ A study by Hirvonen *et al.* compared UV/H_2_O_2_ and activated carbon for tetrachloroethylene (TeCE)-contaminated ground water (*e.g.*, *E*_EO_ (see eqn (8) and (9))), finding that the cost of UV/H_2_O_2_ was on par with carbon adsorption.^80^ Operating costs attributed to electrical energy and hydrogen peroxide depend on the target purification level.

Swaim *et al.* (2011) conducted a comprehensive cost analysis of the UV/H_2_O_2_ process compared to other commercial technologies for treating seasonal taste and odor.^81^ They evaluated six treatment scenarios, including ozone, biological filtration, UV/H_2_O_2_ AOP, and powdered activated carbon (PAC). The study found that UV advanced oxidation was a lower cost solution for seasonal taste and odor events, with a comparable greenhouse gas footprint to ozone. However, ozonation followed by biological filtration was found to be a lower cost approach.

Ozone-based AOPs are cost-effective for degrading most organic micropollutants, but UV-based AOPs are better for persistent compounds such as NDMA and amidotrizoic acid.^82^ When bromide is present, ozone use can lead to bromate formation, which makes UV-based AOPs a better alternative. UV-based AOPs, particularly UV/H_2_O_2_, can effectively break down a broad range of micropollutants, but they require more energy input. Therefore, for the most effective removal of micropollutants, UV-based AOPs offer a comprehensive and less risky option.

### 7.1. Life-cycle assessment considerations

Beyond direct financial costs, the environmental life-cycle impacts of AOPs are a critical factor in sustainable implementation. Life-cycle assessment (LCA) evaluates the environmental burdens associated with all stages of a technology's life, from chemical production to waste disposal. Recent LCA studies of solar-based AOPs provide valuable insights for drinking water applications^83^

• Energy and carbon footprint: high energy consumption in UV-based systems contributes to indirect carbon emissions, underscoring the need for integrating renewable energy (*e.g.*, solar photocatalysis) to improve LCA outcomes.^83^

• Chemical use and byproducts: the production and transport of oxidants contribute to upstream environmental burdens. Furthermore, the formation of transformation products (*e.g.*, bromate, NDMA) requires additional treatment steps, increasing the overall life-cycle impact. On the other hand, the environmental performance of heterogeneous AOPs is highly sensitive to catalyst reuse. For TiO_2_-mediated photocatalysis, impacts decreased by ∼90% when the catalyst was reused ≥5 times *versus* single-use scenarios. Conversely, photo-Fenton processes incur substantial burdens from acidification (H_2_SO_4_) and neutralization (NaOH), which contributed to >50% of impacts in categories such as terrestrial acidification and fine particulate matter formation. Complete iron sludge removal is essential to avoid ecotoxicity impacts exceeding those of the target micropollutants.^83^

The upstream environmental burden of oxidant production is significant. For instance, adding H_2_O_2_ to TiO_2_ photocatalysis increased environmental impacts by ∼20% across multiple categories without proportional gains in micropollutant removal, suggesting that H_2_O_2_ addition may not be environmentally justified in all cases. Similarly, the formation of transformation products (*e.g.*, bromate, NDMA) requires additional treatment steps, increasing the overall life-cycle impact.^83^

• Infrastructure considerations: compound parabolic collector (CPC) infrastructure contributes modestly (∼1–11%) to total impacts, with reinforcing steel, concrete, and chromium steel being the primary contributors. While infrastructure does not alter technology rankings, its inclusion is recommended for comprehensive LCAs.^83^

## 8. Considerations of AOPs

Access to clean drinking water is a fundamental right. Unfortunately, contaminants in drinking water pose a significant health risk. AOPs have proven highly effective in treating a wide range of organic compounds found in drinking water. However, AOPs can result in the formation of transformation products that may retain toxic potential (*i.e.*, the formation of NDMA from the ozonation of dimethyl sulfoxide),^84,85^ and determining their toxicity is both impractical and unfeasible. Fortunately, biological assays offer a practical solution to assessing the quality of treated drinking water. These assays can be tailored to specific needs based on the type of contaminant and AOPs treatment method used. They are simple, cost-effective, and offer an easy-to-use technique for evaluating the potential bioactivity of transformation products of AOPs. By using biological assays, we can ensure that treated drinking water meets safety standards and that everyone has access to clean, safe drinking water.^85^

## 9. Case study

As this state-of-the-art focus on applying AOPs, it is attractive to highlight the application in large-scale pilot plants. UV-based AOPs like UV/H_2_O_2_ are increasingly used to treat contaminants resistant to ozone or where bromate formation is a concern. Over 50 full-scale UV-based AOP systems are now globally processing over 2.2 billion liters of water daily.^13^ UV oxidation is also being evaluated for treating groundwater and surface water sources in pilot-scale and demonstration projects.

The Siheung DWTP improvement is a component of South Korea's infrastructure plan aimed at delivering top-notch water to both high-tech industries and consumers. In 2017, the original design capacity of 101 000 m^3^ day^−1^ was increased to 129 000 m^3^ day^−1^. The study analyzed the seasonal occurrences of blue algae compound 2-methylisoborneol (2-MIB) and the need for a reliable barrier against protozoans. UV/H_2_O_2_ AOP was found to be the best technology, while xylem's low pressure Wedeco MiPRO light system demonstrated superior economic performance.

**Fig. 10:**
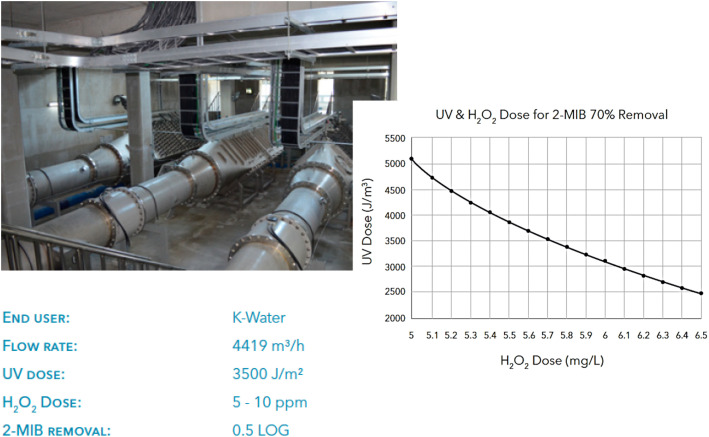
UV/H_2_O_2_ system in the Siheung DWTP, South Korea with its operational parameters and the required UV and H_2_O_2_ doses for 70% removal of 2-MBI.^86^

The Siheung DWTP UV AOP system (Fig. 10) uses three MiPRO photo K-Reactors with 168 Ecoray bulbs, allowing for precise UV dose control for disinfection, taste, and odor compliance. The system's staggered arrangement and ability to dim lamps by up to 50% ensure conformity and energy conservation, ensuring uniformity in UV output. Wedeco's UV-based AOPs control strategy allows for dynamic UV dose adjustment, considering influent water quality. Comprehensive performance tests determined optimal UV radiation and peroxide dosing equilibrium for minimal operational expenses.

## 10. Research gaps and future recommendations

Traditional technologies remove macro parameters but have limited micropollutant removal performance. AOPs can play a crucial role in removing organic pollutants, DBPs caused by chlorination, and disinfection particularly the chlorine resistance protozoa. Therefore, it is recommended to establish AOPs in DWTPs to improve drinking water quality.

The number of research articles on the use of AOPs for wastewater treatment is more than for drinking water treatment. This highlights the significance of using AOPs to eliminate CECs from the source point before they reach the DWTPs.

Homogeneous AOPs are effective at removing NOM, especially the dissolved fraction that can easily pass through filtration systems, which is the primary source of chlorinated DBPs. Homogeneous AOPs are suggested for large-scale DWTPs.

Ozonation facilities should use effective treatment processes to remove ozone DBPs precursors before chlorination. Strategies include controlling bromide sources, optimizing ozone application, chemical adjustments, and pretreatment to reduce bromide/DOM levels. Preventive strategies are crucial for post-ozonation removal.

The O_3_/H_2_O_2_/UV combination is ideal for degrading micropollutant mixtures with multiple contaminants and pathogens, optimizing energy consumption and minimizing harmful by-products like bromate, by leveraging the strengths of each technology.

Fenton and photo Fenton AOPs are very efficient, but because to the low pH requirement and substantial iron sludge formation, they are unsuitable for drinking water treatment; hence, their applications have been limited to wastewater treatment.

The use of UV disinfection units in DWTPs may pose risks if residual chlorine is present, as there is conflicting evidence on whether chlorine photolysis increases or decreases DBPs production. To ensure that residual chlorine does not enter the UV reactor, it is recommended that it be removed or its dose be controlled.

Cold plasma technology offers rapid, low energy, and effective remediation against complex contaminated sites. Commercialization challenges include upscaling and economic factors. Establishing an energy-efficient green cold plasma technique requires practical actions and long-term engineering and industrial criteria.

Heterogeneous AOPs are recommended for small-scale drinking water treatment in decentralized communities and villages. Renewable energy sources like solar photocatalysis can reduce costs and enhance catalyst use. Further research is needed for efficiency and pilot scale testing.

Magnetic separable photo catalysts can be used in slurry systems for drinking water treatment, but there is no pilot plant scale available due to reactor light distribution or electromagnetic unit efficiency issues, necessitating further research on electromagnet separation.

The energy and cost of AOPs for drinking water treatment are site-specific and influenced by target compounds, water quality, and UV transmittance. With AOPs becoming more widespread, more comprehensive economic data is needed. For example, the cost categories should include both capital (equipment, control systems, and buildings) and operating costs (maintenance, energy, chemicals, utilities, labor, analytical services, and so on).

AOPs are effective in disinfecting and treating various organic compounds in drinking water, but the produced by-products and their toxicity can be difficult to determine. Therefore, biological assays are recommended for assessing treated water quality, as they offer a practical solution and can be tailored to specific needs based on the contaminant and treatment method. These assays are simple, cost-effective, and easy to use, evaluating the potential bioactivity of transformation products of AOPs.

Comprehensive economic and LCA data: the energy and cost of AOPs for drinking water treatment are site-specific. With AOPs becoming more widespread, more comprehensive economic and environmental data is needed. Cost categories should include both capital (equipment, control systems, and buildings) and operating costs (maintenance, energy, chemicals, utilities, labor, analytical services). Furthermore, Life-Cycle Assessment (LCA) studies are required to quantify the environmental trade-offs between energy consumption, chemical usage, and sludge disposal.

Finally, when selecting acceptable AOPs, the five previously mentioned factors should be considered: efficiency, energy, reliability, robustness, and ease for implementation.

## 11. Conclusions

Water quality is crucial for preventing infections, reducing health risks, and providing enjoyable drinking water. Conventional treatment methods, such as aeration, filtration, and disinfection, are ineffective in removing micropollutants, necessitating the adoption of AOPs for clean drinking water production. AOPs are effective drinking water treatment technologies that effectively degrade micropollutants, NOM, DBPs, and inactivate bacteria and viruses. However, their complex chemistry affects their applications, design, operations, and limitations.

The number of articles on the use of AOPs for wastewater treatment is higher than for drinking water treatment, highlighting the need to eliminate contaminants from the source before reaching DWTPs. However, scaling up AOPs presents challenges. Research mainly focuses on developing new applications, with heterogeneous systems being more widely published due to their ability to separate the catalyst from treated water effluent. Renewable energy sources like solar photocatalysis could reduce treatment costs and increase water industry interest in AOPs.

However, homogeneous AOPs are recommended for large-scale DWTPs, particularly the O_3_/H_2_O_2_/UV combination, which is ideal for degrading micropollutant mixtures containing multiple contaminants and pathogens while optimizing energy consumption and minimizing harmful byproducts such as bromate by leveraging the strengths of each technology.

In case of DWTPs that were using ozone treatment, prior to chlorination, ozone DBP precursors should be removed using effective treatment techniques. To minimize bromide/DOM levels, strategies include managing bromide sources, improving ozone application, making chemical modifications, and performing treatments. Preventive methods are essential for post-ozonation removal.

In addition, Fenton and photo Fenton AOPs are efficient but not suitable for drinking water treatment due to low pH and iron sludge formation. UV disinfection in DWTPs may pose risks with residual chlorine, so removal or dose control is recommended. Moreover, CAP technology offers rapid, low-energy remediation for complex contaminated sites, but commercialization challenges include upscaling and economic factors.

Heterogeneous AOPs and renewable energy sources are recommended for small-scale drinking water treatment particularly fixed bed photocatalytic reactors. Magnetic separable photo catalysts can be used in slurry systems for drinking water treatment, but pilot plant scales are limited due to reactor light distribution and electromagnetic unit efficiency issues.

In terms of financial considerations, AOPs require comprehensive economic data for site-specific energy and cost categories. While, the formation of oxidation byproducts and understanding their possible toxicity are two significant study areas for AOPs that will assist influence their broad use in the drinking water industry. As a result, biological assays are recommended for testing treated water quality since they provide a practical solution that can be customized to individual requirements based on the contaminant and treatment method.

Finally, in the process of determining appropriate AOPs, it is crucial to take into account the aforementioned five factors: efficiency, energy consumption, reliability, robustness, and implementation simplicity.

## Conflicts of interest

There are no conflicts to declare.

## Data Availability

The data that support the finding of this study are available within the article.
